# Blood–Brain Barrier Permeability: Is 5-Hydroxytryptamine Receptor Type 4 a Game Changer?

**DOI:** 10.3390/pharmaceutics13111856

**Published:** 2021-11-03

**Authors:** Guillaume Becker, Sylvia Da Silva, Amelia-Naomi Sabo, Maria Cristina Antal, Véronique Kemmel, Laurent Monassier

**Affiliations:** 1 Laboratoire de Pharmacologie et Toxicologie NeuroCardiovasculaire UR7296, Département Universitaire de Pharmacologie, Addictologie, Toxicologie et Thérapeutique, Centre de Recherche en Biomédecine de Strasbourg (CRBS), Université de Strasbourg, 1 Rue Eugène Boeckel, CEDEX, 67085 Strasbourg, France; sylvia.dasilva@unistra.fr (S.D.S.); amelia-naomi.sabo@chru-strasbourg.fr (A.-N.S.); laurent.monassier@unistra.fr (L.M.); 2 Pôle Pharmacie-Pharmacologie, Hôpitaux Universitaires de Strasbourg, Avenue Molière, CEDEX, 67098 Strasbourg, France; 3 Laboratoire de Biochimie et Biologie Moléculaire, Hôpitaux Universitaires de Strasbourg, Avenue Molière, CEDEX, 67098 Strasbourg, France; 4 Faculté de Médecine, Institut d’Histologie—Service Central de Microscopie Électronique, Équipe IMIS—ICube UMR7357, Université de Strasbourg, 4 Rue Kirschleger, CEDEX, 67085 Strasbourg, France; mc.antal@unistra.fr; 5 Unité de Fœtopathologie—Service de Pathologie, Hôpitaux Universitaires de Strasbourg, 1 Place de l’Hôpital, 67000 Strasbourg, France

**Keywords:** serotonin, 5-HT_4_ receptor, prucalopride, blood–brain barrier permeability, hCMEC/D3, microvascular endothelial cells

## Abstract

Serotonin affects many functions in the body, both in the central nervous system (CNS) and the periphery. However, its effect on the blood–brain barrier (BBB) in separating these two worlds has been scarcely investigated. The aim of this work was to characterize the serotonin receptor 5-HT_4_ in the hCMEC/D3 cell line, in the rat and the human BBB. We also examined the effect of prucalopride, a 5-HT_4_ receptor agonist, on the permeability of the hCMEC/D3 in an in vitro model of BBB. We then confirmed our observations by in vivo experiments. In this work, we show that the 5-HT_4_ receptor is expressed by hCMEC/D3 cells and in the capillaries of rat and human brains. Prucalopride increases the BBB permeability by downregulating the expression of the tight junction protein, occludin. This effect is prevented by GR113808, a 5-HT_4_ receptor antagonist, and is mediated by the Src/ERK1/2 signaling pathway. The canonical G-protein-dependent pathway does not appear to be involved in this phenomenon. Finally, the administration of prucalopride increases the diffusion of Evans blue in the rat brain parenchyma, which is synonymous with BBB permeabilization. All these data indicate that the 5-HT_4_ receptor contributes to the regulation of BBB permeability.

## 1. Introduction

The blood–brain barrier (BBB) is the most important exchange surface between the blood and the central nervous system (CNS). As the CNS requires a precise and balanced microenvironment to work efficiently, this physical border enables cerebral homeostasis, which regulates the very selective passage of molecules. Microvascular endothelial cells, which constitute the BBB, have specific characteristics, such as the expression of specific transporters causing a selective transcellular passage, a low proportion of transport vesicles (compared to other endothelia), thus limiting the passage of molecules with high molecular weight, and the presence of tight junctions with high electrical resistance [1]. This BBB function is generally considered to be a protective mechanism against virus and unwanted substances circulating in the blood and to control the flow of substances required for brain function [2]. However, BBB failures are described in numerous diseases of the CNS with neurodegenerative, inflammatory, infectious, or even neoplastic components. These dysfunctions can be either seen as a consequence of disease progression or can be involved in the early pathophysiology of the disease (e.g., multiple sclerosis). The consequence is a change in the permeability of the BBB and the delivery of substances from the vascular circulation to the brain or cellular infiltration across the BBB. A better understanding of the mechanisms controlling BBB permeability will help to target BBB interventions that could have therapeutic interests, such as increasing drug delivery to the brain or reducing the damaging effects of inflammation or waste accumulation.

Among the substances that have been studied for their ability to modulate cerebral blood flow and BBB permeability, serotonin (5-HT) has been proposed by several authors [3]. The suggested mechanism is based on an interaction between astrocytes and endothelial cells constituting the BBB. For these authors, cerebral 5-HT acting on astrocytes could lead to the synthesis of ATP and prostaglandins with effects on vascular tone and endothelial permeability [3]. On the other hand, platelets transport 5-HT in the blood and constitute the main store of peripheral 5-HT. During epileptic seizures, it was demonstrated that platelets degranulation modulates BBB permeability and increases cerebral 5-HT concentration [4]. However, a direct role of 5-HT in modulating BBB permeability has not yet been demonstrated. Furthermore, only a few publications have reported that microvascular endothelial cells express mRNA encoding for 5-HT receptors, 5-HT_1D_ and 5-HT_7_ being the most extensively studied, although their functional role in the permeability of the BBB has not been investigated [5]. 

The 5-HT_4_ receptor is highly expressed in neurons of the olfactory bulb, islands of Calleja, basal ganglia, nucleus accumbens, hippocampus, and substantia nigra [6,7,8]. It is co-localized on cholinergic (stimulation of acetylcholine release), glutamatergic and spiny neurons [9]. The 5-HT_4_ receptor is also expressed in intrinsic primary afferent neurons, motor neurons and enterocytes of the gut, in the myocytes of the atria (increased expression in ventricles of heart failing patients) and on the surface of cholinergic and purinergic neurons innervating the detrusor muscle [10]. Finally, its expression is also found in the zona glomerulosa and zona fasciculata of the adrenal gland cortex [11,12].

The 5-HT_4_ receptor is a G protein coupled receptor positively coupled to adenylate cyclase in neurons and enterocytes. Protein kinase A activation leads to the inhibition of voltage-gated K^+^ channels, including Ca^2+^ activated K^+^ channels, produces a long-lasting inhibition of K^+^ channels and activates or inhibits GABAergic synaptic transmission [13]. In cardiac myocytes, 5-HT_4_ receptors activate L-type currents via PKA. After 5-HT_4_ receptors stimulation, cAMP directly activates hyperpolarization-activated current I_h_. These receptors also activate the exchange factor “Exchange Protein Activated by cAMP”, which is a Rap guanine nucleotide exchange factor [14]. Another coupling pathway is described in primary neurons, where the 5-HT_4_ receptor activates the extracellular regulated kinase (ERK) pathway in a G(s)/cAMP/PKA independent manner [15]. This last effect appears to be dependent on the Src tyrosine kinase [15]. 

In this study, we demonstrate the presence of the 5-HT_4_ receptor in human and rat microvascular endothelial cells. Further, hCMEC/D3 cells were used as a cellular model of the BBB, and we hypothesized that 5-HT_4_ receptor stimulation could induce a change in BBB permeability. Therefore, we initiated a study dedicated to characterize the functional roles of this receptor and the signaling pathway stimulated by prucalopride, a 5-HT_4_ receptor agonist, in our cellular BBB model. Finally, the role of the 5-HT_4_ receptor in modifying BBB permeability was studied in prucalopride treated rats.

## 2. Materials and Methods

### 2.1. Materials

The human cerebral microvascular endothelial cells (hCMEC) line, D3 clone (hCMEC/D3; #CLU512-A), were purchased from Tebu-bio company (Le Perray-en-Yvelines, France). Products purchased from Thermo Fisher Scientific (Illkirch-Graffenstaden, France) were as follows: chemically defined lipid concentrate (# 11905031), fetal bovine serum (#10270-106), penicillin–streptomycin (#15070-063), HEPES (#15630-080), TRIzol™ reagent (#15596026), FITC dextran (3 kDa) (#D3305), FITC dextran (10 kDa) (#D1821) and competitive immunoassay kit for the quantification of cyclic AMP (cAMP) (#EMSCAMPL).

The chemicals purchased from Sigma-Aldrich (St. Quentin Fallavier, France) were hydrocortisone (#H0135), ascorbic acid (#A4544), βFGF (#F0291), prucalopride (#SML-1371), GR113808 (#G5918), dimethyl sulfoxide (#D5879), PP2 (#P0042), Trypan blue (#T8154), blue dextran (5 kDa) (#90008) and CD31 monoclonal anti-rabbit antibodies (clone EP78, monoclonal). H89 dihydrochloride (#2910) was purchased from Tocris (Noyal Châtillon sur Seiche, France). The iScript™ RT-qPCR Sample Preparation Reagent, the SYBR Green PCR Master Mix and the PCR primers of 5-HT_4_ receptor and 18S were obtained from Bio-Rad (Marnes-la-Coquette, France) and described in Table S1, Supplementary Materials. PhosSTOP™ (#4906845001) and cOmplete™ ULTRA Tablets (#5892970001) were obtained from Roche (Basel, Switzerland). Specific primary antibodies against 5-HT_4_ receptor (#ab60359) were purchased from Abcam (Cambridge, the United Kingdom). Occludin (#PA5-20755) and ZO-1 (#33-9100) antibodies were obtained from Thermo Fisher Scientific. ERK1/2 (#9102S) and Phospho ERK1/2 (#04797) were obtained from Cell Signaling (Leiden, Netherlands) and Merck Millipore (Molsheim, France), respectively. Claudin-5 (#E-AB-30946) antibody was obtained from Elabscience (distributor Euromedex, Souffelweyersheim, France). Alexa-488 and -563 fluorophore-conjugated secondary antibodies were purchased from Merck Millipore. Goat serum (#S2000-100) was obtained from Dutscher (Bernolsheim, France). Luminata, the ECL reagent, was obtained from Merck Millipore. Vectashield mounting medium containing DAPI (#H-1200), the anti-rabbit biotinylated secondary antibody (#BA-1100), ABC amplification kit (#PK-6100) and VIP substrate (#SK-4600) were purchased from Vector Laboratories (Burlingame, CA, USA).

The 3-(4,5-Dimethylthiazol-2-yl)-5-(3-carboxymethoxyphenyl)-2-(4-sulfophenyl)-2H-tetrazolium (MTS) assay (#ab197010), the Protein Kinase A (PKA) activity kit (#ab139435) and phosphorylated Src and total Src kit (#ab207461) were obtained from Abcam.

### 2.2. Cell Culture

Human cerebral microvascular endothelial cells (hCMEC/D3) were maintained in endothelial basal medium 2 (EBM-2) as previously described [16]. All culture media were supplemented with chemically defined lipid concentrate (1%), fetal bovine serum (5%), penicillin–streptomycin (1%), hydrocortisone (1.4 µM), ascorbic acid (5 µg·mL^−1^), HEPES (10 mM) and βFGF (1 ng·mL^−1^). Tebu-bio provided a vial of this cell line with a passage number between 25 and 27. Cells were cultured and passed twice a week in a 75 cm^2^ flask. 

### 2.3. Cells Treatments

Cells were seeded at 2.2 × 10^6^ cells per cm^2^, on different supports depending on the experiment. Prucalopride and GR113808 were dissolved in dimethyl sulfoxide to obtain 1 mM stock solutions and diluted in the culture medium prior to cell treatment. Endothelial cells were treated for 96 h with prucalopride (10 µM), GR113808 (1 µM) or the combination of prucalopride (10 µM) and GR113808 (1 µM). All cells were depleted of fetal bovine serum at day 3 after the start of treatment, while the treatment was continued.

### 2.4. RNA Extraction and Real Time qPCR

Total RNA was extracted from hCMEC/D3 using the Invitrogen™ TRIzol™ reagent. RNA concentration was quantified by the NanoDrop™ spectrophotometer (Thermo Fisher Scientific, Waltham, MA, USA). Furthermore, 200 nanograms of RNA was extracted to synthesize cDNAs in a thermal cycler with the iScript™ RT-qPCR Sample Preparation Reagent, and then PCR was performed with the SYBR Green PCR Master Mix using a CFX96 Real-Time System (Bio-Rad, Hercules, CA, USA). The relative amount in each sample was normalized to the level of expressed 18S mRNA.

### 2.5. Western Blot

After treatment, the cells were lysed with lysis buffer supplemented with protease and phosphatase inhibitors, cells extracts were collected and centrifuged for 10 min at 10,000× *g* at 4 °C. Total cell lysates containing 10 µg of protein were separated using 10% SDS-PAGE and transferred onto a polyvinylidene fluoride (PVDF) membrane. Non-specific binding sites were blocked with 10% non-fat milk in phosphate buffered saline (PBS) with 0.1% Tween 20 detergent. Membranes were incubated with primary antibodies (5-HT_4_ receptor, ZO-1, occludin, claudin-5, ERK1/2, and Phospho-ERK1/2) overnight at 4 °C. Membranes were extensively washed and then incubated for 1 h with secondary antibodies at room temperature. After another extensive wash, protein bands were visualized by Luminata, ECL reagent, and using the ChemiDoc XRS+ detection system (Bio-Rad). Proteins extracted from human neuroblastoma cell line (SH-SY5Y cell line) were used as a positive control of 5-HT_4_ receptor protein expression [17], and proteins extracted from lung papillary adenocarcinoma (NCI-H441 cell line) were used as a negative control of 5-HT_4_ receptor protein expression. 

The relative intensity was densitometrically determined by using the Image Lab software (Biorad) as the intensity of the bands relative to the total protein ratio revealed by the stain free method. The stain free imaging technology uses a proprietary polyacrylamide gel chemistry to make proteins fluorescent directly in the gel with a short photoactivation, allowing immediate visualization of proteins at any point during electrophoresis and blotting. This method eliminates the inherently problematic use of housekeeping proteins as loading controls on Western blots, permitting the user to obtain truly quantitative Western blot data by normalizing the bands to the total protein in each lane.

### 2.6. Cells Immunofluorescence Assay

Cells were seeded on glass coverslips, fixed with 4% paraformaldehyde (PFA) for 20 min at room temperature. Cells were permeabilized with Triton X-100 at 0.1% and blocked with 10% goat serum in PBS. After a first incubation with rabbit polyclonal anti-5-HT_4_ receptor primary antibody (1:1000) at 4 °C overnight, cells were extensively washed and then incubated with Alexa 488 fluorophore-conjugated secondary antibodies (1:1000) for 4 h. After another wash, coverslips were mounted, and images were obtained with a Leica TCS SP5 MP confocal microscope.

### 2.7. Histological Analysis

In rats, PBS was infused via the left ventricle to initiate rinsing the vasculature and remove blood components. This was followed by 4% PFA in 0.1 M PBS infusion. Brains were fixed by immersion in PFA. After 48 h, the tissue was dehydrated in a series of ethanol solutions of increasing concentration followed by immersion in toluene. Brains were finally placed in liquid paraffin to form a “paraffin block”.

Immunostaining was performed in formalin-fixed, paraffin-embedded rat CNS sections. Thick tissue slices (3–5 µm) were cut using a microtome. After deparaffinization, tissue rehydration and inactivation of endogenous peroxidase, the sections were subjected to the antigen retrieval process (1:1000 rabbit polyclonal anti-5-HT_4_ receptor) at 4 °C overnight, followed by a blocking step to remove non-specific binding sites. The 5-HT_4_ receptor was visualized by peroxidase reaction products.

Paraffin-embedded human brain tissue was analyzed. For this, 5 µm-thick sections were deparaffinized and antigen microwave retrieval in 0.01 M sodium citrate buffer (pH 6) was performed (10 min, 850 W) followed by sequential incubation with rabbit polyclonal anti-5-HT_4_ (1:100 dilution) and Alexa 488-anti-rabbit secondary antibody (1:400 dilution), then with rabbit monoclonal anti-CD31 (1:50) followed by Alexa 568 anti-rabbit secondary antibody (1:400 dilution). Incubation with primary antibodies was performed overnight; secondary antibodies were incubated for 1 h. Extensive washing in 0.01 M Tween was performed between each incubation step. After washing, slides were mounted in Vectashield mounting medium containing DAPI. The specificity of the secondary antibodies was controlled by switching the correspondence with the primary antibodies and by replacing primary antibodies with non-specific immunoglobulins. The 5-HT_4_ immunostaining was equally performed using an anti-rabbit biotinylated secondary antibody, ABC amplification kit and VIP substrate. 

### 2.8. Trans-Endothelial Electric Resistance and Permeability Assay

Cells were seeded at 1 × 10^6^ cells on polyethylene terephthalate inserts with 0.4 µm pore size and 4.5 cm^2^ area (#833930041, Sarstedt, Nümbrecht, Germany) previously coated with a thin layer of collagen I. The hCMEC/D3 cells cultured at 37 °C in 5% CO_2_ constituted our in vitro BBB model. Trans-endothelial electric resistance (TEER) indicating the paracellular permeability of the hCMEC/D3 monolayer was measured with the Evom2^®^ epithelial Ohm meter (World Precision Instruments, Sarasota, FL, USA). The inserts were placed in a cell culture cup chamber for TEER measurement with EndOhm^®^ containing a pair of concentric electrodes. An insert without hCMEC/D3 cells, used as a blank control, was measured and subtracted from the TEER values measured with hCMEC/D3 cells. The result was multiplied by the total membrane surface area to obtain the resistance value in Ω·cm^2^. Measurements were performed daily for 4 days after the start of prucalopride, GR113808 or prucalopride + GR113808 treatments. Kinetics of paracellular permeability were assessed by the area under the curve of TEER (AUC_TEER_) measurement during the 96 h of treatment. When AUC_TEER_ decreased, the hCMEC/D3 barrier permeability increased.

To assess the size of pores that allowed paracellular permeability of the hCMEC/D3 cell monolayer, we used molecules of various molecular weights. Trypan blue (2.9 × 10^−1^ mM, 0.87 kDa), blue dextran (2 mM, 5 kDa) or FITC dextran (10 µM, 3 kDa and 10 µM, 10 kDa) were added in the upper chamber, and their cell monolayer crossing was assessed by reading the absorbance in the bottom chamber. A percentage was obtained as a ratio of absorbance at 595 nm or fluorescence at 490/520 nm, 8 h after the addition of markers in the upper chamber (control set at 100%). When blue dyes were used, samples were analyzed using the iMark™ microplate absorbance reader. Fluorescein was analyzed with a Xenius XM microplate spectrofluorometer reader (Safas, Monaco). The results were expressed as permeability flux percentages. 

### 2.9. MTS Assay

The MTS assay was used to evaluate cell proliferation and viability after prucalopride treatment. The hCMEC/D3 cells were seeded in 96-well culture plates in culture medium at 37 °C for 96 h in the presence or absence of prucalopride (10 µM). The viability was estimated using MTS reduction to the formazan dye. MTS was added to cells and incubated for 2 h. Then, the absorbance of medium containing formazan was measured at 490 nm. The results were expressed as a percentage of viability, compared to the control conditions arbitrarily set at 100%.

### 2.10. Enzyme-Linked Immunosorbent Assay (ELISA)

Competitive immunoassay was performed for the relative quantification of cAMP. The PKA activity analysis was performed with an enzyme-linked immuno-absorbent assay that utilizes a specific synthetic peptide as a substrate for PKA and a polyclonal antibody that recognizes the phosphorylated form of the substrate. Phosphorylated Src and total Src were quantified with an ELISA kit. All these kits were used according to manufacturer’s instructions.

### 2.11. Evans Blue Diffusion to the Brain

BBB permeability in rats was evaluated by the cerebral diffusion of Evans blue after prucalopride or vehicle treatment. All animal care and procedures were in accordance with institutional guidelines and European regulations. The protocol was adapted from Goldim et al. and submitted to the French regulatory authorities and ethics committee (registration number: 26564-202007101135885) [18]. Two groups of 5 adult Wistar rats (control group 301.4 ± 35.84 g; prucalopride group 343.6 ± 36.54 g) (Wistar Han IGS, Charles River, Wilmington, MA, USA) were treated with either daily intraperitoneal injection of 10 mg·kg^−1^ prucalopride or vehicle (10% DMSO in NaCl 0.9%). The animals were monitored in order to detect weight lost, signs of dehydration or diarrhea and each measurable discomfort. Furthermore, 96 h after the start of treatment, 3 mL·kg^−1^ of 2% Evans blue was infused in the left ventricle immediately followed by 10 mL·kg^−1^ of PBS. The brain slices were snaped with a Leica macroscope (M651). Brain samples were homogenized, using a tissue grinder containing 250 µL of 50% orthophosphoric acid per gram of sample, and absorbance at 595 nm (iMark™ microplate absorbance reader, Bio-Rad) was used for a quantitative assessment of blue dye content in the brain parenchyma.

### 2.12. Statistical Analysis

Values were mean ± SEM from three or more independent experiments. Data were reported as mean ± SEM, and *p* < 0.05 was considered statistically significant. Differences were analyzed using a Student’s t-test or one-way ANOVA with GraphPad Prism 6 (GraphPad, San Diego, CA, USA).

## 3. Results

### 3.1. Localization of the 5-HT_4_ Receptor in Human Blood–Brain Barrier

**Figure 1:**
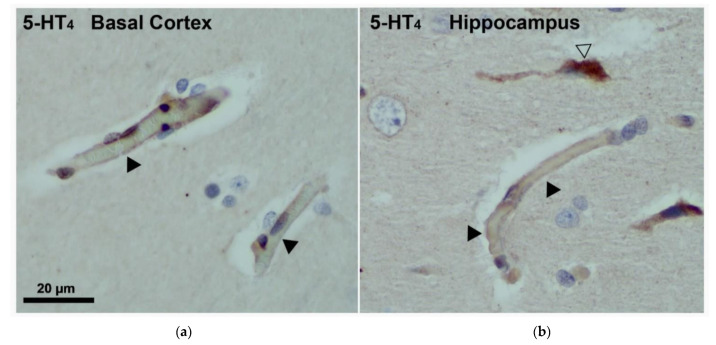
Photomicrograph illustration of 5-HT_4_ receptor immunohistochemistry analysis in human brain (**a**) shows capillary vessels stained with 5-HT_4_ receptor antibody in the basal cortex and (**b**) in hippocampus. A 5-HT_4_ labeling is present on endothelial cells of capillary vessels (◀) and neurons (△).

**Figure 2:**
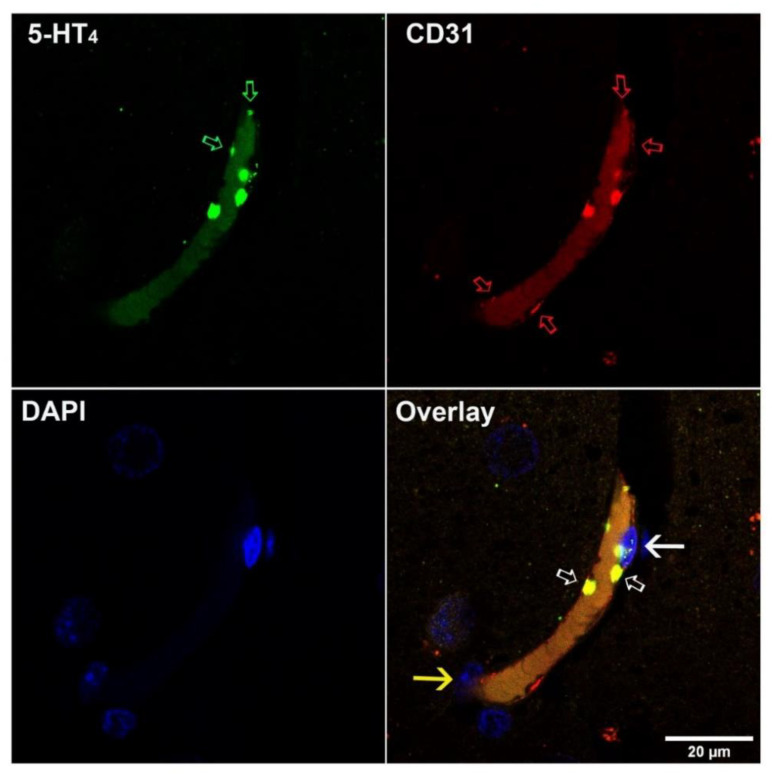
Confocal photomicrographs illustrating the colocalization of 5-HT_4_ receptor and CD31 proteins in the human hippocampus. 5-HT_4_ receptor (in green, Alexa 488, ⇨) and CD31 (in red, Alexa 568, ⇨) colocalize on the endothelial cells of capillary vessels and platelets (⇨). White full arrow indicates the nucleus of an endothelial cell; yellow arrow indicates a pericyte. The slight staining, in green and red, in the middle of the vessels, is due to the autofluorescence of hemoglobin.

Immunostaining was performed in the human brain. The 5-HT_4_ receptor was visualized by immunohistochemistry and immunofluorescence in human brain slices. According to the pictures shown in Figure 1, in the human basal cortex (Figure 1a) and hippocampus (Figure 1b), the 5-HT_4_ receptor was expressed in endothelial cells of capillary vessels. The 5-HT_4_ receptors were also identified in neurons and in platelets as shown in the Figure 1 and Figure 2.

These localizations were confirmed by the double labeling of the 5-HT_4_ receptor and CD31 protein in immunofluorescence assays (Figure 2). CD31, also named platelet endothelial cell adhesion molecule-1 (PECAM-1), is a cell adhesion and signaling receptor that is expressed on hematopoietic cells, including platelets, and on endothelial cells. Endothelial cells were labeled by the 5-HT_4_ receptor and by CD31 antibodies. However, the two proteins labeling did not show complete overlapping, the extent of CD31 staining being superior to the 5-HT_4_ receptor one. Photomicrographs of immunofluorescence controls are shown in Figure S1.

### 3.2. Expression of the 5-HT_4_ Receptor in a Human Blood–Brain Barrier Model (hCMEC/D3)

**Figure 3:**
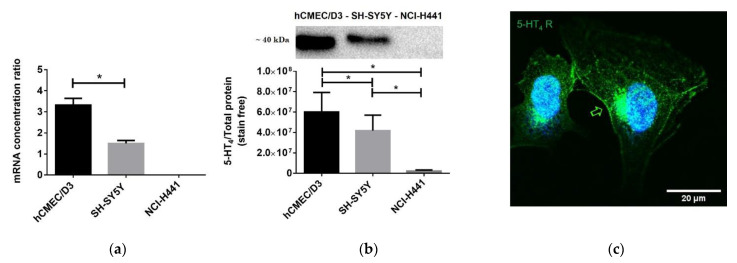
5-HT_4_ receptor expression in hCMEC/D3 cell line: (**a**) real time qPCR quantification of 5-HT_4_ receptor mRNA. (**b**) Western blot analysis of 5-HT_4_ receptor expression. The mRNA and proteins extracted from human neuroblastoma cell line (SH-SY5Y) and lung papillary adenocarcinoma cell line (NCI-H441) were used as positive and negative controls of 5-HT_4_ receptor expression, respectively. (**c**) Photomicrographic illustration of the 5-HT_4_ receptor immunofluorescence analysis in hCMEC/D3. The immunoreactivity of the 5-HT_4_ receptor exhibits a strong distribution in the cytoplasm and on the membrane or perimembrane area. Histograms represent mean ± SEM from three independent experiments. * *p* < 0.05.

Real time qPCR, Western blot and immunofluorescence analysis revealed the presence of the 5-HT_4_ receptor mRNA and protein in the hCMEC/D3 cell line. mRNA transcripts were 2.2-fold higher in the hCMEC/D3 cell line than in the SH-SY5Y cell line (*p* = 0.0062). In the same way, protein expression was 1.4-fold higher in hCMEC/D3 cells (*p* = 0.0443). As shown in Figure 3, this receptor was expressed in hCMEC/D3 cells (controls are shown in Figure S2). The subcellular immunoreactivity was consistent with the endoplasmic reticulum and Golgi involvement in endogenous synthesis of 5-HT_4_ receptor and with its addressing to the cytoplasm, to the membrane or to the perimembrane area. 

### 3.3. 5-HT_4_ Receptor Stimulation Permeabilizes the Blood–Brain Barrier

TEER was used as a marker of BBB integrity and as a measure of paracellular (but not transcellular) permeability and transport. TEER is a widely accepted quantitative technique to measure the integrity of tight junction dynamics in BBB models. The values of TEER can be performed in real time without inducing any cell damage [19].

**Figure 4:**
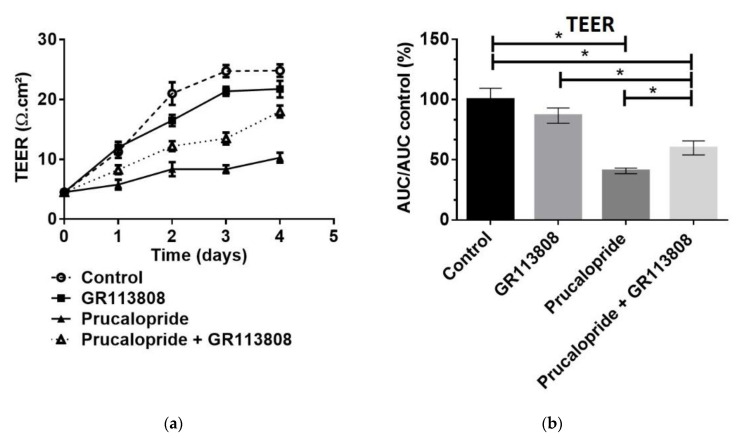
Prucalopride effect on blood–brain barrier TEER in hCMEC/D3 cells. All cells are depleted of fetal bovine serum at day 3 after the start of treatment. Cells are treated with prucalopride, GR113808 or GR113808 + prucalopride for 96 h. (**a**) Daily measured TEER shows a constant increase in TEER in control cells. Treatment with prucalopride for 96 h decrease the TEER attesting the blood–brain barrier opening when GR113808 alone does not change the TEER compared to control. (**b**) The AUC_TEER_ calculated from 0 to 96 h of treatment indicate a significant decrease after prucalopride treatment. GR113808 prevents the effect of prucalopride. Histograms represent mean ± SEM from three independent experiments. * *p* < 0.05; TEER: trans-endothelial electric resistance; AUC: area under the curve.

By measuring the TEER on hCMEC/D3 cells daily, we observed a progressive increase in trans-endothelial resistance in control cells, indicating the formation of a tighter BBB, ideal for permeability studies. However, an increased paracellular permeability was observed upon treatment with 10 µM of prucalopride but not with 1 µM of GR113808 nor with the association of 10 µM of prucalopride + 1 µM of GR113808 (Figure 4a). After a 96 h treatment with prucalopride 10 µM, GR113808 1 µM and prucalopride 10 µM + GR113808 1 µM, the paracellular permeability of the hCMEC barrier was assessed by the area under the curve of the TEER measurement (AUC_TEER_). As shown in Figure 4b, AUC_TEER_ was reduced after treatment with prucalopride (41%, *p* < 0.0001), compared to the control arbitrarily set at 100%. GR113808 alone had no effect on the hCMEC barrier permeability measured by AUC_TEER_ (87%, *p* = 0.268) but significantly reduced the effect of prucalopride on permeability without completely preventing it (60%, *p* = 0.0103).

**Figure 5:**
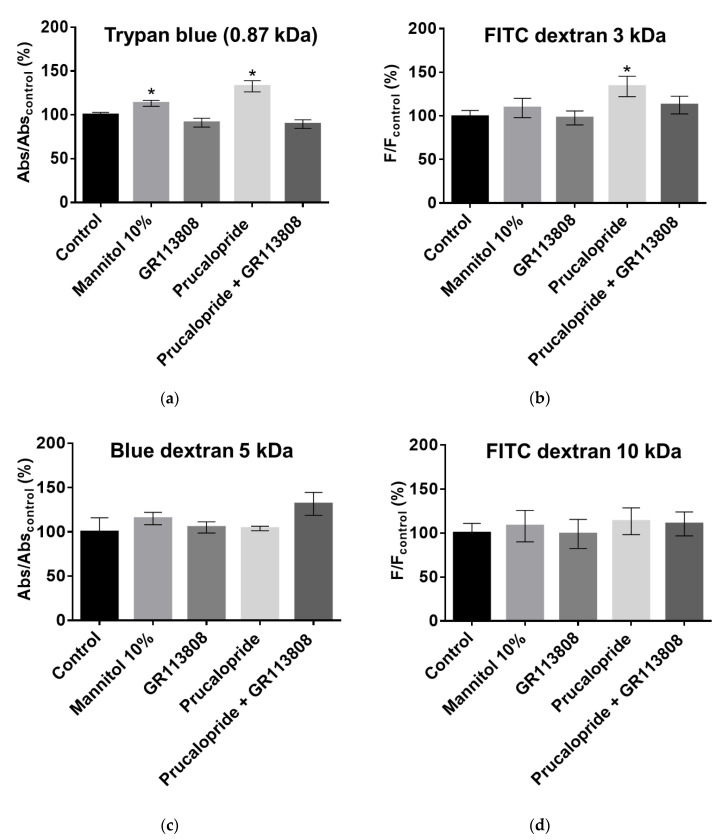
Prucalopride effect on blood–brain barrier permeability in hCMEC/D3 cells. Hypertonic mannitol was used as positive control of blood-brain barrier paracellular permeabilization. Prucalopride stimulation increases the permeability flux of (**a**) Trypan blue and (**b**) FITC dextran 3 (kDa), whereas GR113808 and GR113808 + prucalopride do not change the flux compared to the control. Prucalopride does not change the permeability of (**c**) blue dextran (5 kDa) or (**d**) FITC dextran (10 kDa). Histograms represent mean ± SEM from three independent experiments. * *p* < 0.05; Abs: measure of absorbance; F: measure of fluorescence.

Using colored markers, we quantified the size of molecules that can cross, by paracellular passage, the barrier of hCMEC cells permeabilized by prucalopride. Mannitol was used as a positive control of BBB paracellular permeabilization, due to its hypertonic properties. As shown in Figure 5a,b, cells exposed to 10 µM prucalopride increased the ability of Trypan blue dye (0.87 kDa) or FITC dextran (3 kDa) to cross the cell layer. Prucalopride increased the flux of Trypan blue dye and FITC dextran (3 kDa) by 33% (*p* = 0.018) and 34%, (*p* = 0.0275), respectively. GR113808 did not change the flux compared to the control, but significantly prevented the effect of prucalopride (89.4% and 87.6% compared to control, *p* = 0.0932 and *p* = 0.301, respectively). However, prucalopride did not change the permeability of blue dextran (5 kDa) and FITC dextran (10 kDa), compared to the control (Figure 5c,d), attesting the increased paracellular permeability of the BBB for molecules with molecular weights up to 3 kDa but lower than 5 kDa. 

An MTS assay was used to confirm that cell integrity, due to cell death or proliferation, did not give rise to flux modifications (Figure S3). Absorbance at 490 nm was 1.30 and 1.25 for prucalopride and the control, respectively. Prucalopride did not alter cell viability, compared to the control (*p* = 0.58) and, therefore, such an effect could not explain the prucalopride-induced increase in permeability of the in vitro BBB model.

### 3.4. 5-HT_4_ Receptor Stimulation Modifies the Occludin Expression

**Figure 6:**
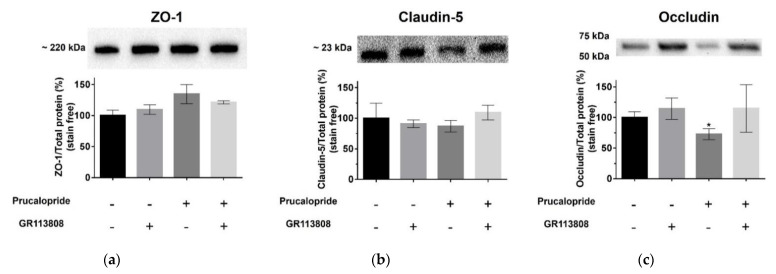
Western blot analysis showing the involvement of the 5-HT_4_ receptor in the regulation of the expression of tight junction proteins in hCMEC/D3 cells. Cells were treated with GR113808 (1 µM), prucalopride (10 µM) or GR113808 (1 µM) + prucalopride (10 µM) for 96 h. Relative integrated density values of (**a**) ZO-1, (**b**) claudin-5 and (**c**) occludin are shown. No correlation is found between the treatment and the expression of ZO-1 and claudin-5. A significant negative correlation between treatment with prucalopride and the expression of occludin is apparent, compared to control. Histograms represent mean ± SEM from three independent experiments. * *p* < 0.05.

To investigate whether the effect of the 5-HT_4_ receptor stimulation on BBB permeability was mediated by a modification of the tight-junction protein expression, a 96 h treatment of cells with prucalopride (10 µM), GR113808 (1 µM) and prucalopride (10 µM) + GR113808 (1 µM) was performed. ZO-1 (Figure 6a), claudin-5 (Figure 6b) and occludin (Figure 6c) expression were analyzed in hCMEC/D3 cells by Western blot experiments. All cells were depleted of fetal bovine serum 24 h before the experiments and treated a second time with the different compounds. Our results showed that ZO-1 and claudin-5 expressions were not significantly different after 5-HT_4_ receptor stimulation (135% and 87%, respectively, compared to control, *p* = 0.2794 and *p* = 0.7395), as shown in Figure 6a,b. In contrast, occludin protein was significantly reduced in cells treated with prucalopride (73% compared to control, *p* = 0.0008) (Figure 6c), and this effect was prevented by GR113808 (115% compared to control, *p* = 0.6822).

### 3.5. 5-HT_4_ Receptor Signaling Pathways Involved in the Blood–Brain Barrier Permeabilization 

The 5-HT_4_ receptor stimulation induces a canonical G(s) protein positively coupled to adenylate cyclase and PKA or a non-canonical G-protein-independent Src/ERK1/2 pathway.

**Figure 7:**
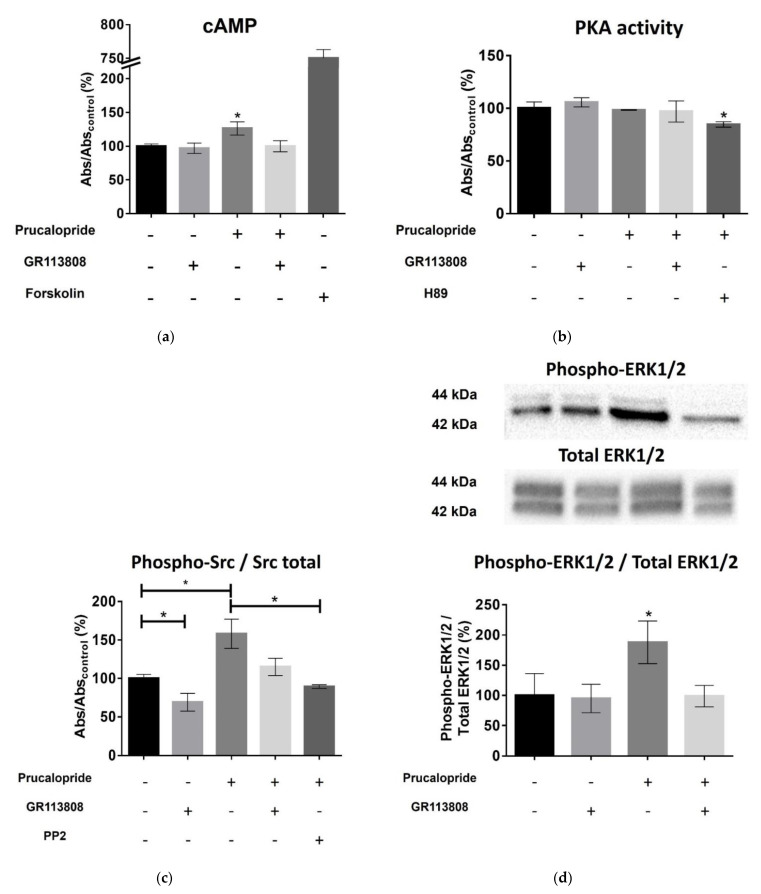
Identification of signaling pathways involved in the 5-HT_4_ receptor effect on the blood–brain barrier. All cells were depleted of fetal bovine serum. (**a**) Stimulation of the 5-HT_4_ receptor by a 15-min long prucalopride (10 µM) treatment results in an increase in intracellular cAMP concentration in the hCMEC/D3 cell line. This increase is prevented by GR113808. (**b**) The increase in cAMP does not translate into a significant increase in PKA activity at 24 h. (**c**) Treatment with prucalopride for 24 h induces Src phosphorylation. This effect is prevented by GR113808 and the Src inhibitor PP2. (**d**) Relative integrated density values of Western blot analysis assessing the effect of 24 h treatment of prucalopride (10 µM), GR113808 (1 µM) and GR113808 (1 µM) + prucalopride (10 µM) on ERK 1/2; phosphorylation in hCMEC/D3 cells show that prucalopride induces ERK 1/2; phosphorylation. Histograms represent means ± SEM from three independent experiments. * *p* < 0.05.

As shown in Figure 7a, stimulation of the 5-HT_4_ receptor by 15 min prucalopride (10 µM) treatment resulted in an increase in intracellular cAMP concentration in the hCMEC/D3 cell line, compared to the control (126.3%, *p* = 0.0238). This increase was fully prevented by GR113808 (100%, *p* = 0.991). However, this cAMP increase did not translate into a significant increase in PKA activity, even after 24 h prucalopride treatment (Figure 7b, 98%, *p* = 0.744). H89, a PKA inhibitor used as the control, decreased PKA activity as expected (85%, *p* = 0.0436). This result was consistent with a constitutive activity of the 5-HT_4_ receptor (or another Gs protein coupled receptor) not correlated with its stimulation. On the other hand, 24 h prucalopride treatment induced Src phosphorylation (Figure 7c, 158%, *p* = 0.0424). This effect was prevented by GR113808 and the Src inhibitor PP2 (115% and 89% in comparison to control, *p* = 0.295 and *p* = 0.139, respectively). Furthermore, 24 h prucalopride treatment was positively correlated with ERK1/2 phosphorylation (Figure 7d, 210.3%, *p* < 0.05). Through 5-HT_4_ receptor stimulation, prucalopride increased the phosphorylation of Src and ERK in the hCMEC/D3 cell line.

### 3.6. Localization of 5-HT_4_ Receptors in Rat Brain Capillaries

**Figure 8:**
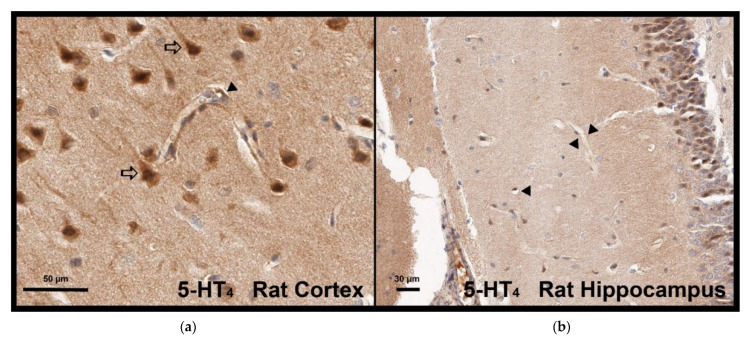
Expression of the 5-HT_4_ receptor by endothelial cells of capillary vessels in the rat cortex (**a**) and hippocampus (**b**). (⇨) indicates neurons with 5-HT_4_ labelling and (◀) indicates endothelial cells of capillary vessels.

Immunostaining was performed, and the 5-HT_4_ receptor was visualized by the brown peroxidase reaction product. According to the immunohistochemistry analyses realized in the rat cortex (Figure 8a) and hippocampus (Figure 8b), the 5-HT_4_ receptor was strongly expressed in neurons and their cellular projections but also in endothelial cells of capillary vessels.

### 3.7. Prucalopride Increases Blood–Brain Barrier Permeability in Rats

**Figure 9:**
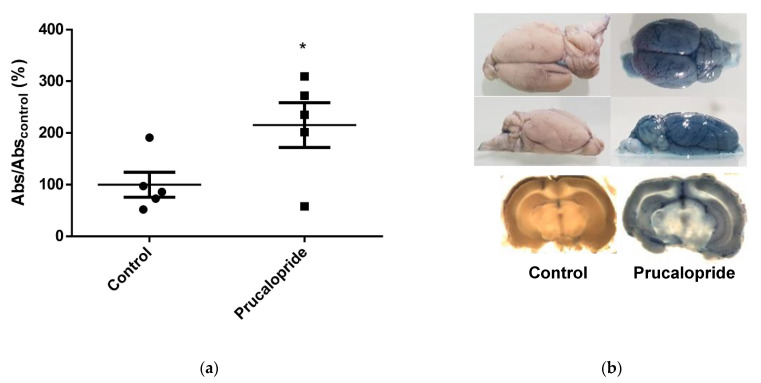
Evaluation of BBB diffusion of Evans blue in brains of Wistar rats. The amount of Evans blue that diffuses into the brain parenchyma increases by the 4-days-long treatment with prucalopride. (**a**) Quantification of the passage of Evans blue in the brain parenchyma by absorbance in rats treated with prucalopride (10 mg·kg^−1^) compared to the vehicle group. (**b**) Photograph of the brains of rats treated with prucalopride after Evans blue infusion compared to the vehicle group. Histograms represent mean ± SEM from 5 rats. * *p* < 0.05. Abs: Absorbance.

Ten adult Wistar rats (average weight = 346 g) were treated daily either by intraperitoneal administrations of prucalopride (10 mg·kg^−1^) or vehicle for 4 days. All treatments were well tolerated by the animals. During experiments, rats did not experiment any discomfort such as weight loss, detectable diarrhea or dehydration. Increased diffusion of the exogenous tracer Evans blue, as an indicator of BBB integrity, was observed in rats after treatment with prucalopride, compared with the vehicle group (Figure 9). The mean absorbance at 595 nm in rat brains, correlating with Evans blue leakage in brain parenchyma, was increased 2.2-fold in the prucalopride group, compared to the control one (*p* = 0.048). 

These experiments confirm that the activation of the 5-HT_4_ receptor expressed in rat microvascularization endothelial cells can drive an increase in BBB permeability.

## 4. Discussion

In this study, through the use of the 5-HT_4_ receptor agonist prucalopride, we investigated the role of the 5-HT_4_ receptor in the modulation of BBB permeability. For the first time, 5-HT_4_ receptor expression was described in the hCMEC/D3 cell line, a well-known in vitro BBB model, but also in human and rat brain microvascular cells [20]. Our second goal was to investigate how prucalopride regulates the paracellular permeability of hCMEC/D3 cells. The 5-HT_4_ receptor stimulation led to a decrease in the TEER of BBB cells model and increased the passage of Trypan blue (0.87 kDa) and FITC dextran (3 kDa) but not blue dextran (5 kDa) and FITC dextran (10 kDa) to the brain. On the other hand, the blockade of the 5-HT_4_ receptor by GR113808, a selective 5-HT_4_ receptor antagonist, prevented the decrease in TEER and the permeability for Trypan blue and FITC dextran (3 kDa). Furthermore, we found that prucalopride affected the expression of the tight junction protein, occludin. Immunoblot experiments revealed that the expression of occludin in cells treated with prucalopride was inferior to that of the controls. However, ZO-1 and claudin-5 tight junction proteins expression was not directly affected by prucalopride. Intercellular tight junctions exhibit a complex molecular architecture involving integral membrane linker proteins, such as occludin and claudins and cytoplasmic adaptor proteins. Occludin, a 65 kDa tetraspan protein, is a key functional component of the tight junction stabilization that plays a critical role in maintaining BBB properties. Occludin C-terminal cytoplasmic domain is required for binding the ZO-1 GLUK domain and the cytoskeleton [21]. Consequently, occludin downregulation could impact the functional role of other tight junction proteins. This phenomenon could explain the tendency to increase the protein expression of ZO-1 observed in our in vitro model after treatment with prucalopride. Some research reported that epithelial barriers are functional in rodents carrying a null mutation of the gene coding for occludin [22]. However, the insertion of an occludin mutant or the null mutation of a gene encoding occludin cannot be compared to the acute effects of prucalopride treatment leading to the downregulation of occludin in a constituted barrier.

The 5-HT_4_ receptor is a G-protein-coupled receptor positively coupled to adenylate cyclase in neurons and enterocytes [13]. In primary neurons, the 5-HT_4_ receptor activates the ERK pathway in a G(s)/cAMP/PKA-independent manner [15]. The 5-HT_4_ receptor-mediated ERK activation is dependent on Src, a nonreceptor tyrosine kinase [15]. Therefore, we investigated the signaling pathways involved in the occludin-mediated permeability variations. Surprisingly, prucalopride moderately improved cAMP production in hCMEC/D3 and did not activate PKA. On the other hand, prucalopride induced Src and ERK1/2 activation. Src kinase has already been described as being involved in occludin regulation. In transient focal cerebral ischemia, Takenaga et al. reported that the inhibition of Src activity decreases the tyrosine phosphorylation of occludin in brain capillaries and attenuates increased permeability of the BBB [23]. Moreover, Zhang et al. described that propofol attenuates the tumor necrosis factor-α induced occludin downregulation by inhibiting ERK1/2 in the hCMEC/D3 cell line [24]. The 5-HT_4_ receptor is a constitutively coupled Gs receptor. According to Liu et al., it can be hypothesized that constitutive activated Gs protein is critical to initiate ERK1/2 activation and that another ligand-induced pathway activation is responsible for the amplification and maintenance of ERK signaling [25].

The microvascular brain endothelial cells restrict blood–brain exchanges. However, this barrier is finely regulated by the surrounding environment, namely, the neurovascular unit. Astrocytes are particularly described for regulating the permeability of BBB [3]. Consequently, co-culture models, including astrocytes, are frequently proposed [26]. However, the characteristics of this heterogeneous lineage depend on the brain structure of origin. In vitro models make complex the discrimination of cellular mechanisms localized in endothelial and other cell types. Although their roles are less studied, other cell lines appear to influence the permeability of the BBB (as pericytes or microglial cells). Thus, in vitro modeling of the BBB cannot be extrapolated in vivo; this is why new animal experiments are necessary. In this work, rather than developing complex coculture to model BBB, we decided to confirm the in vitro results by animal experiments. The quantitative assessment of BBB permeability was performed by measuring the passage of Evans blue in brain structures, a method widely described in the literature [18]. The administration of Evans blue after 4 days of treatment with prucalopride (10 mg·kg^−1^ per day) confirmed the effect of prucalopride on BBB permeability. Barthet et al. reported that 5-HT_4_ receptor mediated ERK activation is transient and that 5-HT_4_ receptor colocalizes with Src even after the receptor endocytosis [15]. Given that rats were sacrificed, we could not assess the duration of BBB opening after the end of treatment. Unlike many studies that assessed the permeabilization of BBB in a localized area of the brain, a localized effect of prucalopride was not expected. Evans blue is homogenously distributed in neuron-rich areas but appears to diffuse less in the white matter, favoring the hypothesis of the heterogeneity of BBB across brain structures [27]. Future work should focus on the origin of 5-HT involved in the modulation of BBB permeability mediated by the 5-HT_4_ receptor. It could be of a peripheral origin and therefore, coming from platelet degranulation (platelets contain about 95% of the 5-HT of the body). A central origin of 5-HT for the effects observed in this study cannot be fully ruled out. 

The 5-HT_4_ receptor is a potential target to develop new pharmacological strategies aiming to open the BBB. Some drugs developed in neurodegenerative or brain tumor diseases show poor cerebral diffusion and require high posology to achieve therapeutic concentrations in the brain. These high dosages may cause peripheral adverse events, poor tolerance or safety issues. New pharmacological strategies that open the BBB without disrupting it are required. These active substances targeting the 5-HT_4_ receptor could transiently enhance brain distribution and consequently improve the efficacy and safety of several drugs.

## 5. Conclusions

In this paper, we show that the 5-HT_4_ receptor is expressed by endothelial cells constituting the BBB. The stimulation of the 5-HT_4_ receptor located on the microvascular brain endothelial cells increased BBB paracellular permeability. In vitro data suggest that increased BBB permeability is induced by Src and ERK1/2 phosphorylation and mediated by occludin tight junction downregulation. In order to ascertain the presence of this molecular mechanism in the living brain, additional in vivo experiments are required.

## Data Availability

The data presented in this study are available on request from the corresponding author.

## Supplementary Materials

The following are available online at https://www.mdpi.com/article/10.3390/pharmaceutics13111856/s1, Table S1: Primer’s sequences used for real time PCR analysis, Figure S1: Photomicrographs of immunofluorescence controls, Figure S2: Photomicrographs of 5-HT_4_ receptor immunofluorescence in hCMEC/D3 cells and controls, Figure S3: MTS assay confirms that prucalopride does not alter cell viability.
