# Supplementary material for: Blood–Brain Barrier Permeability: Is 5-Hydroxytryptamine Receptor Type 4 a Game Changer?
Source: Pharmaceutics. 2021 Nov 3;13(11):1856. doi: 10.3390/pharmaceutics13111856 (PMC8619119; doi:10.3390/pharmaceutics13111856)
Supplement: Supplementary file 1 [file pharmaceutics-13-01856-s001.zip › pharmaceutics-1435503-supplementary.pdf]

pharmaceutics

MDPI

Supplementary Materials: Blood–Brain Barrier Permeability: Is 5-Hydroxytryptamine Receptor Type 4 a Game Changer?

Guillaume Becker, Sylvia Da Silva, Amelia-Naomi Sabo, Maria Cristina Antal, Véronique Kemmel and Laurent Monassier

Table S1. Primer's sequences used for real time PCR analysis.

Gene	Biorad (Reference)	Amplicon Sequence	Amplicon Length
HTR4	qHsaCID0012115	AGAGCAGGTGATGGCGTAGGGCTTGTTGACCATGAAGA-<br>CACAGTACGTAGAGTTAGAG-<br>TTCTGGTTGAACTTCCTCTTTTCTATCAAATCAATTATGCCAAT<br>GTTATTCCAGCCTTGCATTATAGGG	97
18S	qHsaCED0037454	GTGGAACGTGTGATCACCATTATGCAGAATCCACGCCAG-<br>TACAAGATCCCAGACGGTTCTT-<br>GAACAGACAGAAGGATGTAAAGGATGGAAAATACA	67

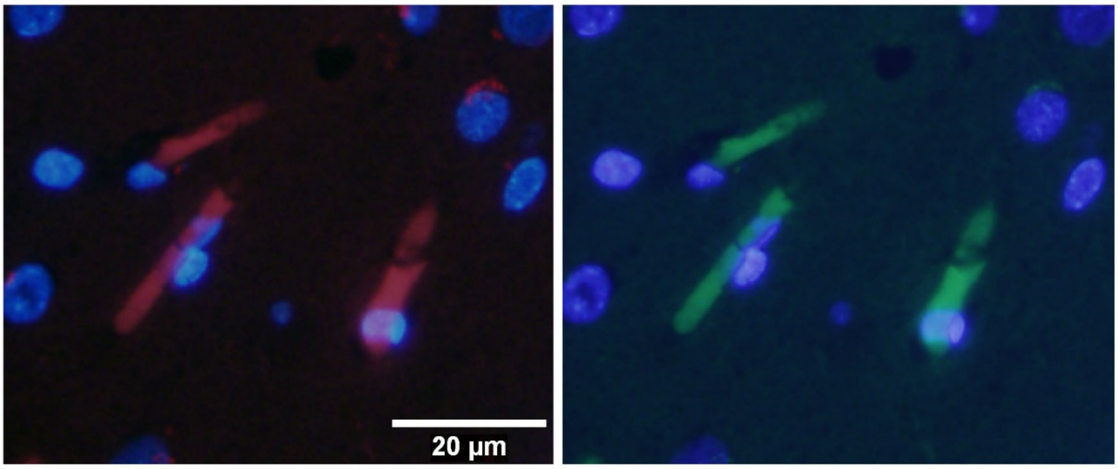

Figure S1. Photomicrographs of immunofluorescence controls. The specificity of the secondary antibodies was controlled by replacing the primary antibodies with non-specific immunoglobins sera. No labelling is observed on the wall of the vessels. The red and green staining in the middle of the vessels is due to hemoglobin autofluorescence. Nuclei are stained in blue by DAPI.

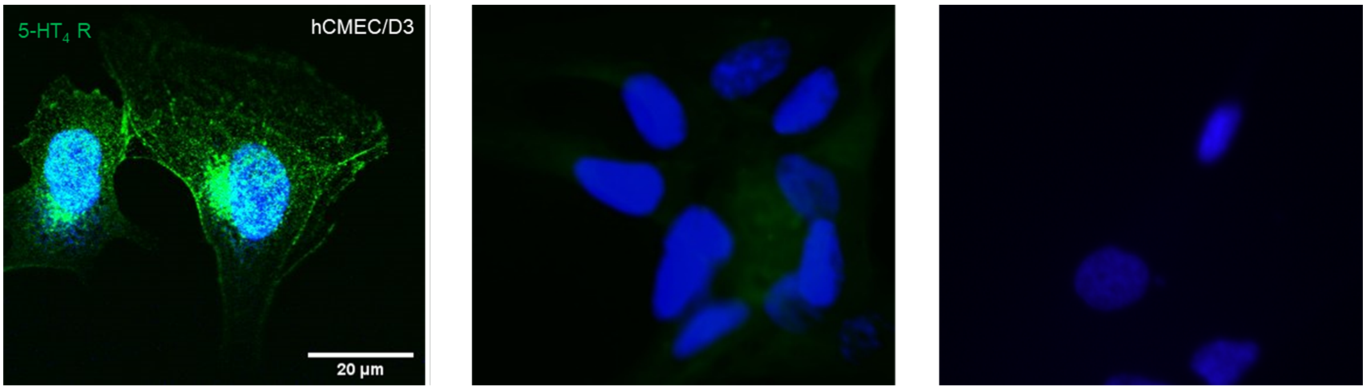

(a) (b) (c)

Figure S2. Photomicrographs of 5-HT$_4$ receptor immunofluorescence in hCMEC/D3 cells and controls. (**a**) Immunofluorescence micrograph obtained with 5-HT$_4$ and secondary antibodies. (**b**) and (**c**) are controls (**b**) Immunofluorescence

micrograph obtained with non-specific immunoglobins sera and with secondary antibodies. (**c**) Immunofluorescence micrograph obtained with the secondary antibody alone.

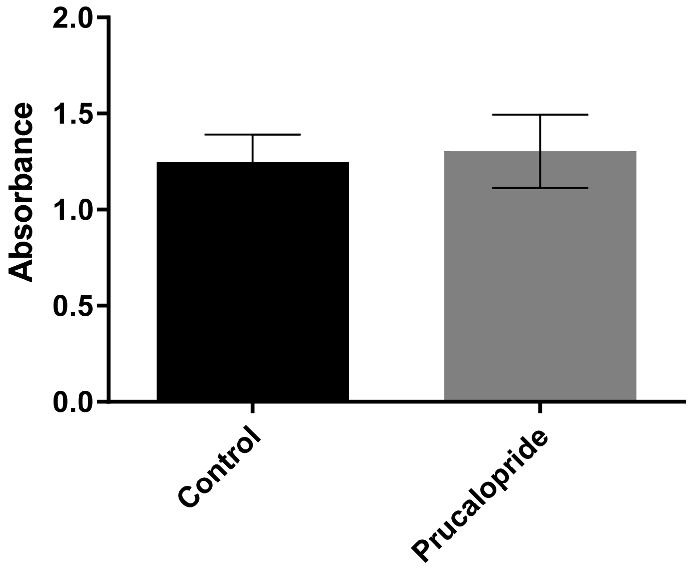

Figure 3. MTS assay confirms that prucalopride does not alter cell viability. No difference is observed at 490 nm for absorbance between cells treated with prucalopride or control. Histograms represent mean ± SEM from six independent experiments.
